# Maize breeding enhances lodging resistance through vertical allocation changes of stem dry matter and nitrogen

**DOI:** 10.3389/fpls.2025.1514045

**Published:** 2025-03-14

**Authors:** Alejo Ruiz, Agustin Listello, Slobodan Trifunovic, Sotirios V. Archontoulis

**Affiliations:** ^1^ Department of Agronomy, Iowa State University, Ames, IA, United States; ^2^ Bayer Crop Science, Chesterfield, MO, United States

**Keywords:** genetic gain, plant breeding, plant height, stalk, lodging

## Abstract

The maize stem provides structural support to other plant organs and stores carbohydrates and nitrogen (N) that can be remobilized to grain. Yet it remains unknown whether breeding programs have indirectly affected vertical stem dry matter and N allocation. Our objectives were to i) determine the dry matter and N allocations across different stem portions in maize hybrids released from 1980 to 2020 at the start (R2) and end of grain-filling period (R6), and ii) investigate the dry matter and N remobilization patterns by stem portion. We studied 23 Bayer Crop Science hybrids (release year 1980 to 2020) of different relative maturities (short and long maturity) in 2021 and 2022. Stem dry weight, N concentration, and N content by stem portion (four equal-length sections) were measured at R2 and R6 stages. We found that the average vertical distribution of the stem dry matter and N content along the plant height was 8% (top), 17% (middle-top), 29% (middle-bottom), and 46% (bottom). Maize breeding slightly reduced the total stem dry matter and N content at R2 stage in both relative maturities, and this reduction occurred in the top two quarters, with no significant change in the bottom stem portion. At R6 stage, the N content was significantly reduced in long maturity hybrids, and all the stem portions contributed to this reduction. Newer hybrids of both relative maturities remobilized less dry matter than the older hybrids (17 vs 20%) but slightly more N (39 vs 36%). We concluded that maize breeding efforts have indirectly affected vertical stem dry matter allocation towards less stem dry matter above the ear leaf. This change reduces plants’ center of gravity, which could explain why new hybrids are more resistant to lodging and can accommodate high plant densities. This study brings new data and knowledge, which enhances our understanding of indirect breeding consequences on maize plant traits.

## 1 Introduction

Over the last decades, maize breeding has significantly increased grain yield by ~100 kg ha^-1^ year^-1^ across different germplasms (Duvick et al., 2004; Abdala et al., 2018; King et al., 2024). In addition to higher yields, maize breeding has reduced the proportion of stems in the total biomass while enhancing ear dry matter, the leaf-to-stem ratio, above-ground dry matter and nitrogen (N) accumulation at maturity, and stem N remobilization (Chen et al., 2015; Mueller et al., 2019; Saenz et al., 2025). Despite these changes, which could potentially increase genotypes susceptibility to lodging, breeding efforts have successfully reduced stalk lodging susceptibility by 0.16% per year (Russell, 1984; Duvick et al., 2004). Stalk lodging can affect biomass accumulation during grain filling and mechanical harvest, leading to an annual yield loss of 5-20% worldwide (Flint-Garcia et al., 2003).

Dry matter and N in the grains can derive from two sources: (i) photosynthesis and N uptake during grain-fill period and (ii) remobilization from stems and leaves. The amount of remobilization varies across growing conditions (environment x management) and genotype choices that determine the grain demand (Daynard et al., 1969; Fairey and Daynard, 1978; Setter and Meller, 1984; Ouattar et al., 1987; Kiniry et al., 1992; Bihmidine et al., 2013; Fernandez et al., 2020). Data from 30 site-year-management combinations in Iowa, USA, showed that maize stems remobilize, on average, 25% of the dry matter and 55% of the N content during grain-fill period (Archontoulis et al., 2020). Leaves typically remobilize more dry matter and N than stems (Li et al., 2019; Fan et al., 2023), while the degree of remobilization has been associated with the amount of dry matter or N at silking time (DeBruin et al., 2013; Fan et al., 2023; Palmero et al., 2024). Furthermore, dry matter and N remobilization are higher under stressful conditions (water, N-stress) than under non-stress (Ouattar et al., 1987; Uhart and Andrade, 1995; Chen et al., 2015; Sekhon et al., 2016; Fan et al., 2023; Palmero et al., 2024).

Maize breeding has increased stem N remobilization with years of hybrid release (from 31 to 58%) but not leaf N remobilization, which has remained at the same levels (around 55%; Mueller et al., 2019). This strategy could maintain the leaves photosynthetically active for a longer period (DeBruin et al., 2017; Mueller et al., 2019; Fernandez et al., 2020). Fan et al. (2023) provided further physiological insight into vertical patterns of dry matter and N remobilization from leaves. They showed that leaves positioned in the middle of the canopy remobilize more dry matter and N to grains than the top or bottom leaves. In contrast to leaves, stems must provide structural support in addition to providing carbohydrates and N to the grain. Information regarding stem dry matter and N allocation and remobilization across the plant height (different stem sections) remains unknown and can provide insight into standability and stem logging resistance.

Maize breeding has successfully decreased susceptibility to stalk lodging while reducing the stem proportion in the total plant biomass and enhancing dry matter partitioning to the ear and N remobilization from the stem. Based on these changes, we hypothesize that maize breeding most likely has reduced the dry matter and N remobilization from the bottom parts of the plant. In this study, we investigate this hypothesis by measuring stem dry matter and N allocation and remobilization patterns at different plant heights (stem portions) in 23 hybrids released from 1980 to 2020. Specifically, our objectives were:

1. To determine stem vertical dry matter and N allocation patterns at the onset and end of the grain-fill period in maize hybrids released from 1980 to 2020.
2. To investigate stem vertical dry matter and N remobilization patterns during the grain-fill period.

Understanding these factors could help explain how breeding programs have successfully developed hybrids that exhibit higher productivity while enhancing stalk resistance. This is particularly challenging given the apparent trade-off between lodging resistance and high productivity in maize (Zhang et al., 2021).

## 2 Materials and methods

### 2.1 Experimental design and crop management

Two on-farm field experiments, one with 12 short maturity maize hybrids (from 100 to 105-day) and one with 11 long maturity maize hybrids (from 110 to 115-day), were conducted in central Iowa, USA, in 2021 and 2022, We evaluated in total 23 Bayer Crop Science maize hybrids commercialized from 1980 to 2020 (**Table 1**). Hybrids were selected based on their commercial relevance and were all conventional with no GMO traits.

**Table 1:** Hybrid name, year of release, and relative maturity (RM) used in the experiments.

Short maturity			Long maturity		
Name	Year	RM	Name	Year	RM
		days			days
T1000	1983	100	T1100	1983	110
LH74+LH59	1986	105	LH74+LH51	1986	110
DK547	1986	105	LH132+LHE136	1986	115
DK527	1994	102	DK604	1994	110
LH198+LH172	1995	102	DK618	1996	111
DK521	1995	102	DKC61-69	2005	111
DKC52-43	2003	102	DKC63-42	2006	113
DKC52-21	2004	102	DKC61-21	2006	111
DKC52-59	2006	102	213-19VT2P	2016	113
DKC52-04	2013	102	DKC51-54	2015	111
DKC55-20	2015	105	DKC60-88	2017	110
DKC54-38	2014	104			

All hybrids were conventional with no GMO traits.

The experimental design was a randomized complete block with two replications. In the short maturity hybrids experiments, the plot size was 15 m^2^ (4-row plot), while in the long maturity hybrid experiments, 60 m^2^ (8-row plot). Maize hybrids were planted at near optimum planting dates for this region (Apr 30 to May 13; Baum et al., 2020), at current plant densities of 7.9 to 8.5 pl m^-2^, and received sufficient N fertilizer application (**Table 2**). Maize hybrids were sown with precision planters used in breeding programs. Experiments were all rainfed, with a row spacing of 76 cm, and the previous crop was soybean.

**Table 2:** Weather data, management practices, and average grain yield across experiments.

Year	Maturity	Location	Weather				Management practices			Grain yield
			Precipitation	Temperature	VPD	Radiation	Plant date	Plant density	N rate	
			mm	°C	kPa	MJ m^-2^	Date	Plants m^-2^	kg N ha^-1^	Mg ha^-1^
2021	Short	Britt, IA	611	19.2	0.80	3173	May 1	7.9	151	13.2
	Long	Fraser, IA	573	19.9	0.84	3208	April 30	8.4	202	14.9
2022	Short	Fraser, IA	537	18.8	0.82	3139	May 13	8.4	202	12.0
	Long	Fraser, IA	537	18.8	0.82	3139	May 13	8.5	202	14.2

Cumulative precipitation, average air temperature, average vapor pressure deficit (VPD), and cumulative solar radiation from April through September. Grain yield data at 150 g kg^-1^ moisture.

### 2.2 Environmental conditions

The soil at all experimental fields was clay loam, rich in soil organic matter (3.4% in 0-30cm) and with a shallow water table (mean 1.3 m, range: 0.3 to 3 m; Archontoulis et al., 2020; Elli and Archontoulis, 2023). Weather conditions for the experiments were provided by Bayer Crop Science (gridded weather dataset ERA5-Land, Muñoz-Sabater et al., 2021). The cumulative growing season (April through September) rain was 591 mm in 2021 and 537 mm in 2022, with the 30-yr average being 677 mm (**Table 2**). Crops experienced 16% below-normal rainfall (**Supplementary Figure S1**), and slightly above normal radiation (<5%) in both years. The mean growing season temperature was 1.3°C above normal in 2021 and around normal in 2022 (normal = 18.6°C). The growing season average vapor pressure deficit (VPD) averaged 0.82 kPa, which was 25% above normal across locations and years.

### 2.3 Plant measurements and calculations

**Figure 1:**
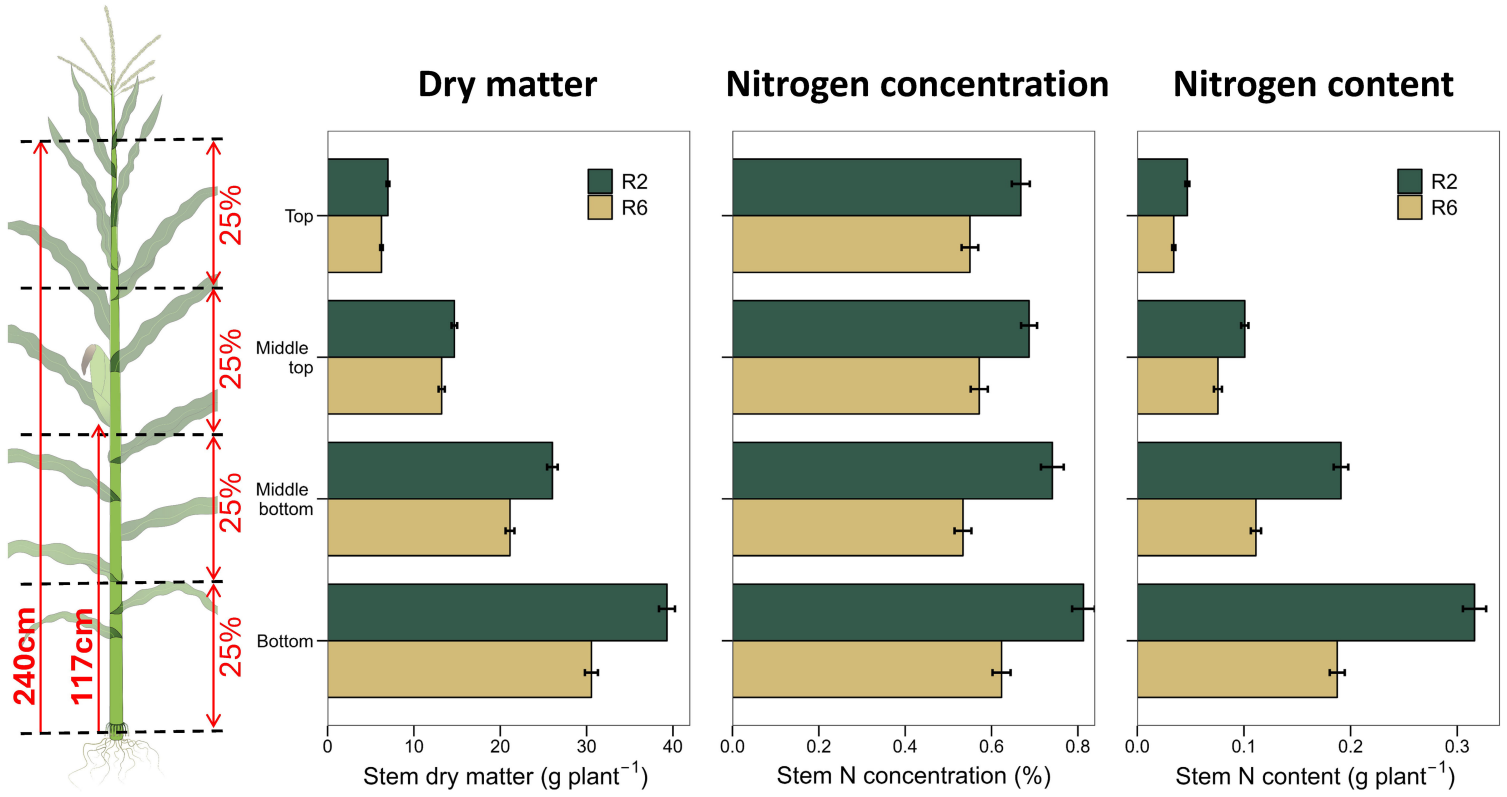
Methodology used to measure vertical stem dry matter, N concentration, and N content (left). Based on plant height, the stem was cut into four equal-length sections. Relative maturities, hybrids, and experiments average data for R2 and R6 stages (right). Bars represent the standard error of the mean. Significant differences exist between R2 and R6 for all traits and stem sections.

Plant counts were determined early in the season, showing an average plant density of 8.3 pl m^-2^ with very small variability among plots within an experiment (average standard deviation of 0.39 pl m^-2^ and coefficient of variation of 4.8%). At the start of grain-fill (R2 stage) and at physiological maturity (R6 stage, Abendroth et al., 2011), one plant was harvested from the center rows of each plot at the ground level. Following plant harvest, the brace roots, leaf blades, ears, and tassel were removed from the plant, and the stem was cut into four equal-length sections (**Figure 1**). Samples were dried in a forced-air oven at 60°C until constant weight and weighed separately. After grounding the samples, stem N and C concentrations were determined using a CHNS Elemental Analyzer (Elementar Americas). Plant and ear heights were measured during plant harvest as the distance from the soil surface to the insertion of the flag leaf blade and from the soil surface to the base of the main ear, respectively. Prior to mechanical harvest, five consecutive ears from the center row were hand harvested for grain N content (CHNS Elemental Analyzer). Plot-level grain yield estimates were derived from the middle rows of each plant using combine harvesters. Grain yield data are reported here at 150 g kg^-1^ moisture, while dry matter and N content data are reported on dry basis.

Stem dry matter (DM) and N remobilization were determined as:


(1) $$\begin{array}{c} Stem\;DM\;remobilization\;(\%)\\ =\frac{Stem\;DM\;at\;R2\;stage\;-\;Stem\;DM\;at\;R6\;stage}{Stem\;DM\;at\;R2\;stage}\;*100 \end{array}$$

(2) $$\begin{array}{c} Stem\;N\;remobilization\;(\%)\\ =\frac{Stem\;N\;at\;R2\;stage\;-\;Stem\;N\;at\;R6\;stage}{Stem\;N\;at\;R2\;stage}\;*100 \end{array}$$

### 2.4 Statistical analysis

Plant trait genetic gain was calculated using a linear fixed-effect model using the *lm* function in R (R Core Team, 2022). We analyzed separately the genetic gain for each trait, stem section, and relative maturity combination. The statistical model included the hybrid year of release, season, replicates within a season, and the interaction hybrid year of release with season as fixed effects. Genetic gain significance (hybrid year of release effect) was tested using a type III F-test (*emmeans* package; Lenth et al., 2023). We found weak evidence of year of release and season interaction across most traits. Thus, a single genetic gain was reported across seasons. Mean comparisons between R2 and R6 stem dry matter, N concentration, and N content were made by paired t-test (**Figure 1**). Regression analyses in R (R Core Team, 2022) were used to explore stem sections dry matter and N remobilization relationships.

## 3 Results

### 3.1 Grain yield, grain N content, and plant and ear height

**Figure 2:**
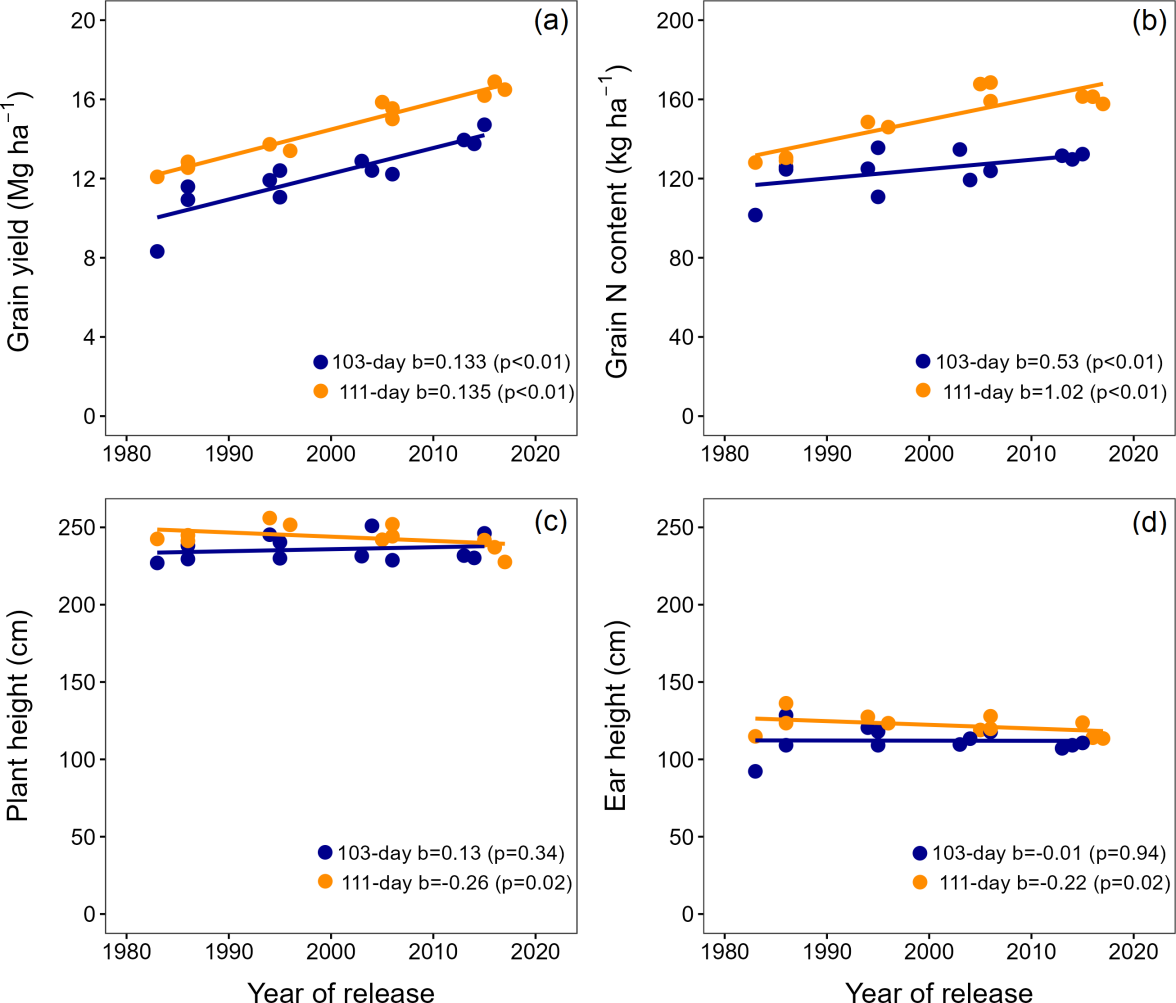
Grain yield **(a)**, grain N content **(b)**, plant height **(c)**, and ear height **(d)** for short and long maturity hybrids released between 1980 and 2020. The points represent the average per hybrid across experiments.

Across experiments and hybrids, the grain yield significantly increased with the year of hybrid release from 8.3 to 16.9 Mg ha^-1^ (**Figure 2a**). Long maturity hybrids had consistently greater yields than the short maturity hybrids. However, the genetic gain was similar across relative maturities (134 kg ha^-1^ year^-1^; **Figure 2a**). The grain N amount significantly increased with the year of hybrid release (**Figure 2b**) but at different rates depending on relative maturity. The grain N content genetic gain in long maturity hybrids was 2-fold greater than that of short maturity hybrids (1.02 vs. 0.53 kg N ha^-1^ year^-1^; **Figure 2b**).

Across all hybrids, the plant height ranged from 227 to 256 cm (**Figure 2c**), the ear height from 92 to 136 cm (**Figure 2d**), and the ear-to-plant height ratio from 0.40 to 0.53 (**Supplementary Figure S2**). In long maturity hybrids, plant and ear heights were significantly (p<0.05) reduced with the year of release by 0.26 and 0.22 cm year^-1^, respectively (**Figures 2c, d**). New long maturity maize hybrids had 9 cm shorter plant height and 8 cm lower ear height compared to old hybrids. The ear-to-plant height ratio did not change with the hybrid year of release in long maturity products. In short maturity hybrids, the plant height, ear height, and ear-to-plant height ratio did not change with the year of release (**Figures 2c, d**; **Supplementary Figure S2**).

### 3.2 Stem dry matter accumulation and allocation

**Figure 3:**
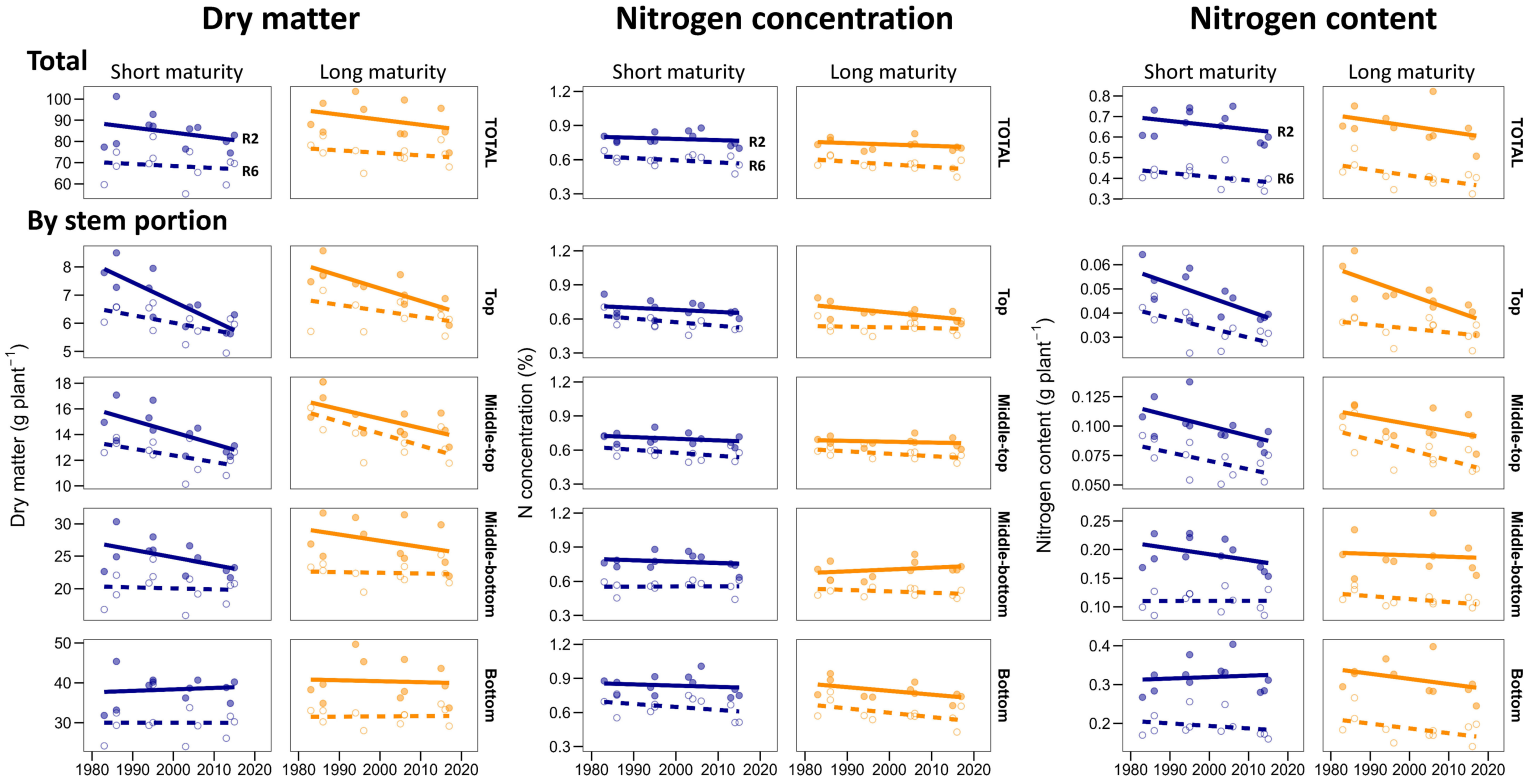
Stem dry matter, N concentration, and N content for stem quarters at the start of grain-fill (R2, solid line) and physiological maturity (R6, dashed lined) in short maturity (blue) and long maturity (orange) hybrids released between 1980 and 2020. The points represent the average per hybrid across experiments. Genetic gains and significance are detailed in **Supplementary Table S1**.

Across hybrids, the total stem dry matter ranged from 75 to 104 g plant^-1^ at R2 stage and from 55 to 83 g plant^-1^ at R6 (**Figure 3**). On average, the whole stem dry matter decreased by 18% from R2 to R6 stage (**Table 3**; **Figure 1**). The average vertical distribution of the stem dry matter along the plant height was: 8% (top), 18% (middle-top), 30% (middle-bottom) and 44% (bottom, **Figure 1**). The relative distribution of dry matter changed little from R2 to R6 stage (**Figure 1**).

**Table 3:** Stem dry matter, N concentration, and N content and remobilization from different stem portions (see **Figure 1**) at R2 and R6 stages for old and new hybrids from each relative maturity group (103 vs 111-day).

	Short maturity						Long maturity					
	1983			2015			1983			2017		
	R2	R6	Remob	R2	R6	Remob	R2	R6	Remob	R2	R6	Remob
			%			%			%			%
Dry matter (g plant^-1^)												
Top	7.9	6.5	18	5.7	5.6	2	8.0	6.8	15	6.5	6.1	6
Mid-top	15.8	13.3	15	12.8	11.6	9	16.5	15.6	5	14.0	12.5	10
Mid-bottom	26.8	20.0	25	23.1	19.7	15	29.0	22.6	22	25.7	22.3	13
Bottom	37.7	29.3	22	38.9	29.5	24	40.8	31.5	23	40.0	31.7	21
**Total**	**88.2**	**69.2**	**22**	**80.6**	**66.4**	**18**	**94.3**	**76.5**	**19**	**86.2**	**72.6**	**16**
Nitrogen concentration (%)												
Top	0.71	0.62	–	0.65	0.52	–	0.72	0.54	–	0.60	0.51	–
Mid-top	0.72	0.62	–	0.68	0.54	–	0.69	0.60	–	0.66	0.53	–
Mid-bottom	0.79	0.55	–	0.75	0.56	–	0.68	0.53	–	0.73	0.49	–
Bottom	0.86	0.69	–	0.82	0.61	–	0.85	0.66	–	0.73	0.53	–
**Total**	**0.80**	**0.63**	**-**	**0.77**	**0.58**	**-**	**0.75**	**0.60**	**-**	**0.71**	**0.52**	**-**
Nitrogen content (g N plant^-1^)												
Top	0.06	0.04	25	0.04	0.03	23	0.06	0.04	37	0.04	0.03	18
Mid-top	0.11	0.08	26	0.09	0.06	28	0.11	0.09	16	0.09	0.07	28
Mid-bottom	0.21	0.11	48	0.18	0.11	36	0.19	0.12	37	0.19	0.10	43
Bottom	0.31	0.20	36	0.32	0.18	44	0.34	0.21	38	0.29	0.17	43
**Total**	**0.69**	**0.44**	**37**	**0.63**	**0.39**	**38**	**0.70**	**0.46**	**34**	**0.61**	**0.37**	**39**

Values are mean estimates derived from the models shown in **Figure 3**.

In both relative maturities, newer hybrids had less total stem dry matter at R2 stage than older hybrids (-0.28 g plant^-1^ year^-1^, *p*>0.05). The overall reduction from 1980 to 2020 hybrids was 10% and occurred in the top three-quarters, with no significant change in the bottom stem portion (**Figure 3**). Because of this, new hybrids had 7% (long maturity) and 13% (short maturity) increased relative allocation of dry matter at the bottom stem portions at R2 stage (**Table 3**). At R6 stage, the patterns were similar to those in R2 stage, with no change in stem dry matter at the bottom with the year of release and a reduction in top-plant stem dry matter, which led to an overall insignificant change in total stem dry weight in both relative maturities (**Figure 3**; **Table 3**).

### 3.3 Stem nitrogen concentration and allocation

Across hybrids, whole stem average N concentration ranged from 0.67 to 0.88% at R2 stage and from 0.45 to 0.68% at R6 stage (**Figure 3**). On average, the whole stem N concentration decreased by 24% from R2 to R6 stage (**Figure 1**; **Table 3**). The average vertical distribution of the stem N concentration was relatively uniform along the plant height at both growth stages, with a slightly higher N concentration at the bottom of the stem at R2 (**Figure 1**; **Table 3**). In long maturity hybrids, whole stem average N concentration was similar between old and new hybrids at R2 stage, but there was a significant decrease of -0.0023% year^-1^ (*p*<0.05) at R6 stage. Most of this reduction was driven by the bottom stem portion (**Figure 3**). In short maturity hybrids, whole stem average N concentration slightly decreased (*p*>0.05) with the year of hybrid release for both R2 and R6 stages (**Figure 3**; **Supplementary Table S1**).

Across hybrids, the total stem N content ranged from 0.51 to 0.82 g N plant^-1^ at R2 stage and from 0.32 to 0.55 g N plant^-1^ at R6 (**Figure 3**). On average, the whole stem N content decreased by 37% from R2 to R6 stage (**Table 3**; **Figure 1**). The average vertical distribution of the stem N content along the plant height was 8% (top), 17% (middle-top), 28% (middle-bottom), and 47% (bottom, **Figure 1**), which is nearly identical to the stem dry matter distribution. The relative distribution of the stem N content along the plant remained unchanged from R2 to R6 stage (**Table 3**; **Figure 1**).

At R2 stage, newer hybrids of both relative maturities had slightly less (*p*>0.05) total stem N content than older hybrids (**Figure 3**). However, different stem portions exhibited different patterns with the year of release: the N content significantly (*p*>0.01) decreased in the top-middle portions of the stem and remained unchanged in the middle-bottom portions at R2 stage (**Figure 3**).

At R6 stage, newer long maturity hybrids had less (*p<0.05*) total stem N content than older hybrids (**Figure 3**). The reduction with the year of release was 2-fold larger for long maturity hybrids compared to short maturity hybrids (0.0027 vs. 0.0015 g N year^-1^, **Figure 3**). All stem portions contributed to this reduction at the R6 stage in long maturity hybrids (**Figure 3**). In short maturity hybrids, the reduction occurred at the top-middle portions of the stem only.

### 3.4 Dry matter and N remobilization

Across hybrids, stems remobilized 18% dry matter and 37% N content from R2 to R6 stages. Newer hybrids of both relative maturities remobilized less dry matter than the older hybrids (20% vs 17%) but slightly more N (39 vs. 36%; **Table 3**).

**Figure 4:**
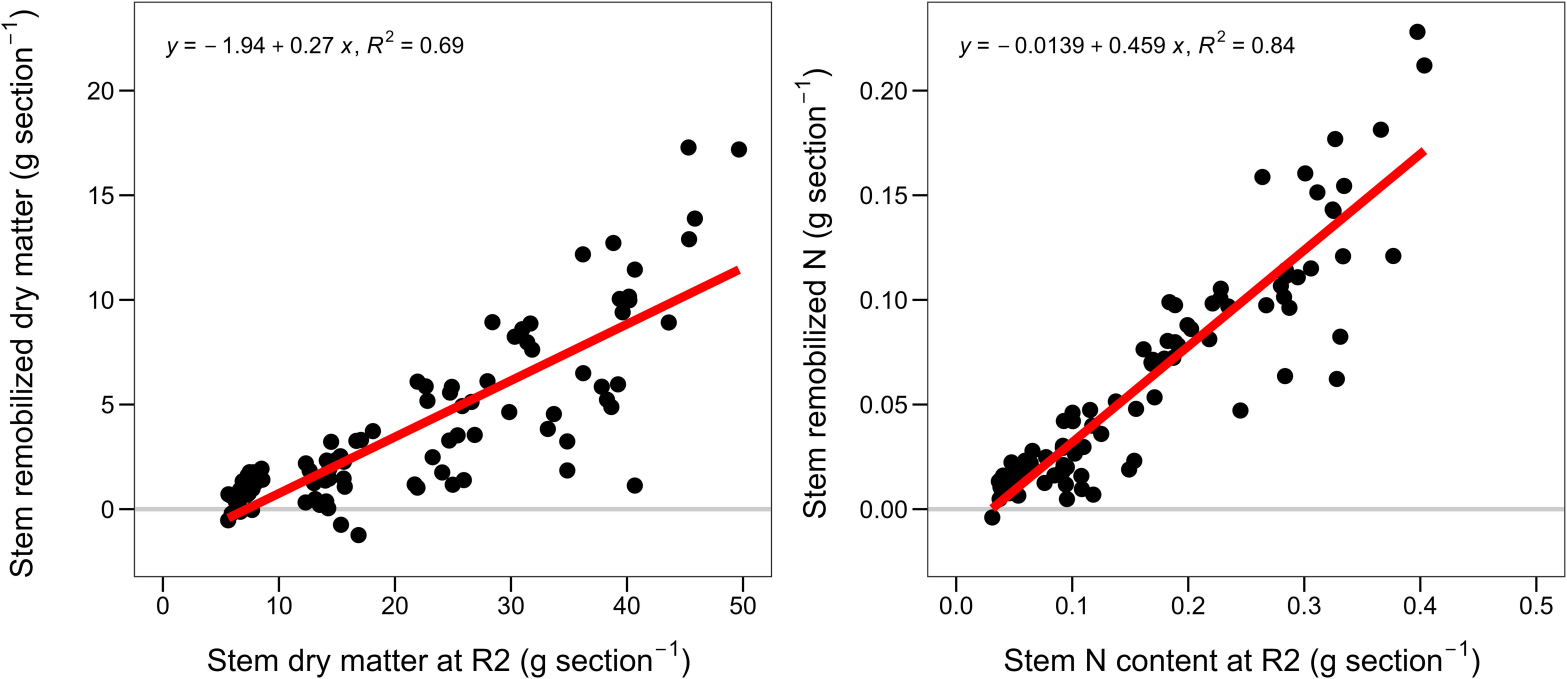
Stem dry matter remobilization versus stem dry matter at R2 (left) and stem N remobilization versus stem N content at R2 stage (right). For stem dry matter and N remobilization see Equations 1 and 2.

Across hybrids and relative maturities, the below the ear leaf stem portions contributed 80% of the total stem dry matter and N remobilization (**Figure 1**; **Table 3**). The stem dry matter and N remobilization per stem portion were positively associated with the dry matter and N content at R2 stage (**Figure 4**). Therefore, bottom stem portions with more dry matter and N contents remobilized the most (**Table 3**). Furthermore, the dry matter and N remobilization patterns were similar across relative maturities and old and new hybrids (**Supplementary Figure S3**). On average, for every 1 g of remobilized dry matter, 0.01g N were remobilized (**Supplementary Figure S4**). In relative terms, we found 20% N remobilization (base) with no dry matter remobilization, and beyond that, for every 1% increase in dry matter remobilization, the N remobilization increased by 0.8% (**Supplementary Figure S4**).

## 4 Discussion

We found that maize breeding has indirectly affected stem dry matter and N allocations and the corresponding remobilization rates (**Figure 3**; **Table 3**). Together with the new data on vertical distribution of stem dry matter and N along the plant height, our findings enhance our understanding of stem dry matter and N dynamics during grain-fill that could help explain why newer hybrids are more resistant to lodging (Russell, 1984; Duvick et al., 2004).

### 4.1 Different vertical dry matter and N distribution between stems and leaves

We found that most of the stem N is allocated at the bottom of the plant due to the greater dry matter content (**Figures 1**, **3**; **Table 3**). This vertical pattern is different from that of leaf N distribution within the canopy, in which the most leaf N is at the middle canopy, around the ear leaf (Hirose and Werger, 1987; Bertheloot et al., 2008; Fan et al., 2023). Furthermore, we showed that most stem dry matter and N remobilization during grain-fill derive from the bottom stem (**Table 3**). Again, this pattern differs from that of leaves, in which the largest amount of leaf dry matter and N remobilization derives from the middle of the canopy (Fan et al., 2023). These differences between stems and leaves vertical patterns are important for further understanding C and N physiological dynamics during grain-fill. Given that most previous studies reported dry matter and N vertical distribution in leaves or leaves and stems combined without focusing on the stem (Johnson et al., 2010; Fan et al., 2023), our study adds new knowledge. Our results that the bottom stem quarter had a slightly greater N concentration (**Figure 1**; **Table 3**) than other stem portions agree with findings by DeBruin et al. (2013), the only study to our knowledge that reported vertical distributions in stem N concentration.

### 4.2 Maize breeding has decreased stem dry matter allocation above the ear leaf at R2

Our study is the first to report maize breeding effects on dry matter and N allocation and remobilization patterns by stem portion. Previous studies have examined maize breeding effects on the total stem dry matter and N in a small number of genotypes of long maturity (Chen et al., 2015; Fernandez et al., 2020). Our finding that the total stem dry matter has been affected little by breeding (**Figure 3**) agrees with the literature (Chen et al., 2015; Saenz et al., 2025). However, we revealed that this small change was not proportional across stem portions and crop stages. The top-plant stem dry matter at R2 stage has been significantly decreased by 16 to 22% in both relative maturities, while the bottom-plant stem dry matter has remained unchanged (**Figure 3**). Because the bottom part of the stem dry matter is large (on average 45% of total, **Figure 1**), no significant breeding effects are evident at the whole stem dry matter. Consequently, the distribution of dry matter within the stem has changed with breeding towards less dry matter at the top-portions of the stem (**Table 3**).

### 4.3 Long season newer hybrids have less stem N concentration at R6 stage

Stem N concentration remained unchanged at R2 stage in both relative maturities. However, at R6 stage, the average stem N concentration has significantly decreased in long maturity (111-day) hybrids in agreement with previous studies (Chen et al., 2015; Fernandez et al., 2020) and has remained unchanged in short maturity hybrids (**Figure 3**). Interestingly, the reduction in long maturity hybrids N concentration was similar across stem sections (**Figure 3**). Considering that stem represents up to 50% of the total maize residue (Bender et al., 2013), breeding probably has reduced maize residue quality (higher CN ratio) in long maturity hybrids, with no effect on short maturity hybrids, which could impact residue decomposition dynamics. The fact that stem N concentration has remained unchanged in short maturity hybrids is important, considering that residue decomposition constraints are more significant in the target environments of this group of hybrids (Sindelar et al., 2013; King et al., 2024).

### 4.4 Breeding had contrasting effects on stem dry matter and N remobilization

Maize hybrids remobilized a 2-fold larger amount of stem N than dry matter (**Table 3**), in agreement with literature (Chen et al., 2015; Archontoulis et al., 2020). Breeding slightly reduced stem dry matter remobilization but slightly increased stem N remobilization in both relative maturities, in agreement with previous findings (**Table 3**; Chen et al., 2015; Mueller et al., 2019). The larger N requirements (grain N content, **Figure 2**) in newer hybrids, together with a lesser stem minimum N concentration boundary (Fernandez et al., 2020), might have resulted in the greater stem N remobilization. In fact, DeBruin et al. (2017) found that grain N was positively associated with hybrid leaf N remobilization at limited N availability. Furthermore, we found that the greater stem N remobilization of the new hybrids is due to greater N remobilization at the bottom of the stem, providing new evidence supporting the greater N use efficiency in newer hybrids (Mueller et al., 2019). Similar to DeBruin et al. (2013) findings for the leaves and Ciampitti et al. (2013) for the shoot, we found that stem sections with more dry matter and N remobilized the most (**Figure 4**). However, we found that stems remobilized only 0.46g N per g N content at R2, in comparison to the 0.63g reported in leaves (DeBruin et al., 2013). These remobilization relationships were consistent across maturities and hybrids year of release (**Supplementary Figure S3**).

This study does not account for management effects such as N fertilizer rate and plant density, which can affect N remobilization (Uhart and Andrade, 1995; Fan et al., 2023). Previous era studies found that genotypic differences in N content and remobilization between hybrids released in different years were only evidenced under sufficient/high N availabilities (Haegele et al., 2013; DeBruin et al., 2017; Fernandez et al., 2020). Besides, Chen et al. (2015) found that plant density treatments did not affect stem N remobilization. Therefore, the breeding effects on dry matter and N allocation and remobilization reported in this study might have been more moderate if hybrids had been tested under N-limiting conditions.

### 4.5 Stem dry matter allocation might explain lodging resistance in newer hybrids

Newer hybrids support greater ear biomass and remobilize more N from the stem into the grain (**Figure 2**; Mueller et al., 2019; Ruiz et al., 2023; Saenz et al., 2025), characteristics that make a crop more susceptible to lodging. However, newer hybrids are more resistant to lodging (Duvick and Cassman, 1999). Lodging resistance is typically related to plant architecture, stem node diameter and internode length, stem N remobilization, synthesis of lignin and cellulose, and disease tolerance (Rajcan and Tollenaar, 1999; Jackson-Ziems et al., 2014; Bian et al., 2016; Xue et al., 2016; Ahmad et al., 2018; Stubbs et al., 2023). However, Xue et al. (2020) showed that most genotypic differences in resistance to stalk lodging were explained by plant and ear height, stem dry weight, and average dry weight per unit length of the bottom nodes. In addition, Zhang et al. (2021, 2023) evaluated hybrids with different lodging susceptibility and found that lodging-resistant hybrids had lower plant and ear height and partitioned more dry matter to the stem base. Our study showed that new long maturity plants were shorter and had lower ear height (**Figure 2**), even though the ear-to-plant height ratio did not change with the hybrid year (**Supplementary Figure S3**). Besides, we found, for both maturities, a reduction in the dry matter allocation to the top stem sections with the hybrid year or release (**Figure 3**), increasing the relative contribution of the bottom quarter to the total stem dry matter (**Table 3**). This change in dry matter allocation reduces the height of the plants’ center of gravity, which could explain why newer hybrids are more resistant to lodging. Future research could investigate the long-term effects of breeding on other traits associated with lodging resistance, such as lignin and cellulose content in the stem.

## 5 Conclusion

We concluded that maize breeding efforts for greater yields have indirectly affected vertical stem dry matter and N allocation and remobilization patterns. Newer hybrids allocate less stem dry matter above the ear leaf than old hybrids in both relative maturities. The allocation of dry matter to the bottom portions of the stem remained unchanged with the year of hybrid release in both maturities. This change in vertical dry matter allocation along the stem reduces plants’ center of gravity, which could explain why new hybrids are more resistant to lodging and can accommodate increased plant densities compared to old hybrids. Maize breeding decreased the stem N concentration only in long maturity hybrids at R6 stage without affecting vertical distribution. The lesser stem N concentration implies lower residue quality in the new hybrids, which may affect residue decomposition patterns. The stem N remobilization was nearly doubled (37%) compared to dry matter remobilization (18%), and its distribution along the plant height was more uniform than that of dry matter. Maize breeding slightly reduced stem dry matter remobilization but increased stem N remobilization in both relative maturities. Overall, this study brings new data and knowledge, enhancing our understanding of historical yield increases and indirect consequences on maize plant traits.

## Data Availability

The raw data supporting the conclusions of this article will be made available by the authors, without undue reservation.
