# Supplementary material for: Maize breeding enhances lodging resistance through vertical allocation changes of stem dry matter and nitrogen
Source: Front Plant Sci. 2025 Mar 14;16:1514045. doi: 10.3389/fpls.2025.1514045 (PMC11949881; doi:10.3389/fpls.2025.1514045)
Supplement: Supplementary file 1 [file DataSheet1.docx]

**SUPPLEMENTARY MATERIALS**

**Supplementary material**

| **Table S1:** Genetic gain (b) and significance for dry matter, N concentration, and N content across stem portions at R2 and R6. | | | | | | | | | | |
| --- | --- | --- | --- | --- | --- | --- | --- | --- | --- | --- |
| **Relative maturity** | | **Short maturity** | | | | **Long maturity** | | | | |
| **Stage** | | **R2** | | **R6** | | **R2** | | | **R6** | |
| **Trait** | **Position** | **b** | **p-value** | **b** | **p-value** | **b** | **p-value** | **b** | | **p-value** |
|  |  |  |  |  |  |  |  |  | |  |
| Dry matter | TOTAL | -0.2393 | 0.187 | -0.0865 | 0.633 | -0.2401 | 0.256 | -0.1133 | | 0.408 |
| (g) | Top | **-0.0681** | **0.000** | **-0.0281** | **0.050** | **-0.0446** | **0.001** | -0.0207 | | 0.144 |
|  | Second | **-0.0920** | **0.005** | -0.0531 | 0.089 | **-0.0739** | **0.045** | **-0.0898** | | **0.008** |
|  | Third | -0.1156 | 0.055 | -0.0102 | 0.860 | -0.0962 | 0.189 | -0.0070 | | 0.887 |
|  | Bottom | 0.0367 | 0.674 | 0.0051 | 0.955 | -0.0243 | 0.826 | 0.0047 | | 0.940 |
|  |  |  |  |  |  |  |  |  | |  |
| N concentration | TOTAL | -0.0010 | 0.506 | -0.0016 | 0.333 | -0.0012 | 0.337 | **-0.0023** | | **0.046** |
| (%) | Top | -0.0018 | 0.285 | -0.0031 | 0.059 | **-0.0035** | **0.014** | -0.0006 | | 0.630 |
|  | Second | -0.0014 | 0.248 | **-0.0026** | **0.032** | -0.0007 | 0.476 | **-0.0021** | | **0.025** |
|  | Third | -0.0012 | 0.494 | 0.0001 | 0.934 | 0.0016 | 0.291 | -0.0012 | | 0.304 |
|  | Bottom | -0.0011 | 0.631 | -0.0026 | 0.322 | -0.0033 | 0.091 | **-0.0038** | | **0.073** |
|  |  |  |  |  |  |  |  |  | |  |
| N content | TOTAL | -0.0021 | 0.240 | -0.0015 | 0.328 | -0.0028 | 0.160 | **-0.0027** | | **0.037** |
| (g) | Top | **-0.0006** | **0.001** | **-0.0004** | **0.005** | **-0.0006** | **0.000** | -0.0002 | | 0.206 |
|  | Second | **-0.0008** | **0.009** | **-0.0007** | **0.012** | **-0.0006** | **0.041** | **-0.0008** | | **0.002** |
|  | Third | -0.0010 | 0.114 | 0.0001 | 0.828 | -0.0002 | 0.722 | -0.0005 | | 0.191 |
|  | Bottom | 0.0004 | 0.705 | -0.0006 | 0.579 | -0.0013 | 0.247 | -0.0012 | | 0.132 |
|  |  |  |  |  |  |  |  |  | |  |


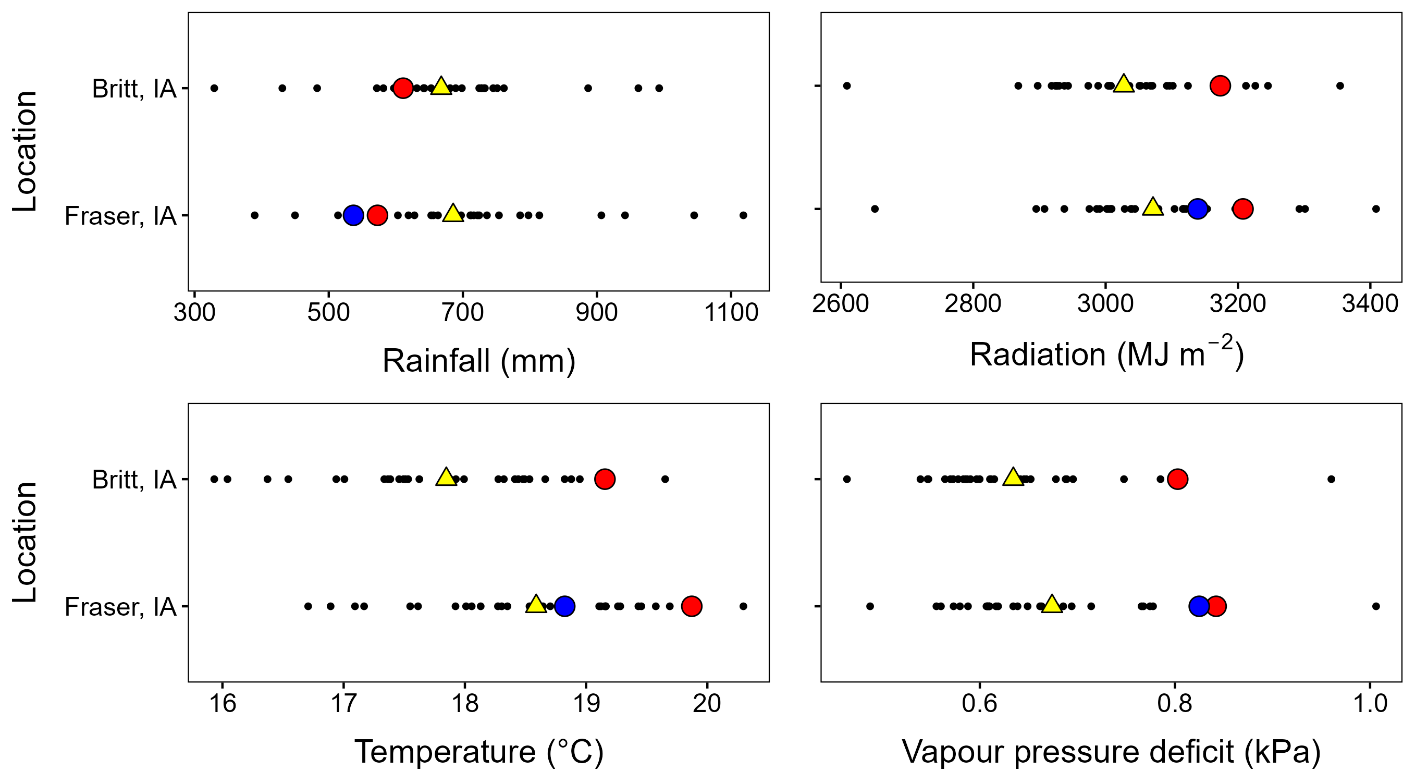


**Fig. S1.** Historical, 2021 growing season, and 2022 growing season weather summaries, including cumulative rainfall, cumulative radiation, average temperature, and average vapor pressure deficit from April through September. Red dots represent the values for the 2021 growing season, blue dots for the 2022 growing season, yellow triangles for the historical average, and black dots for the last 30 growing seasons.


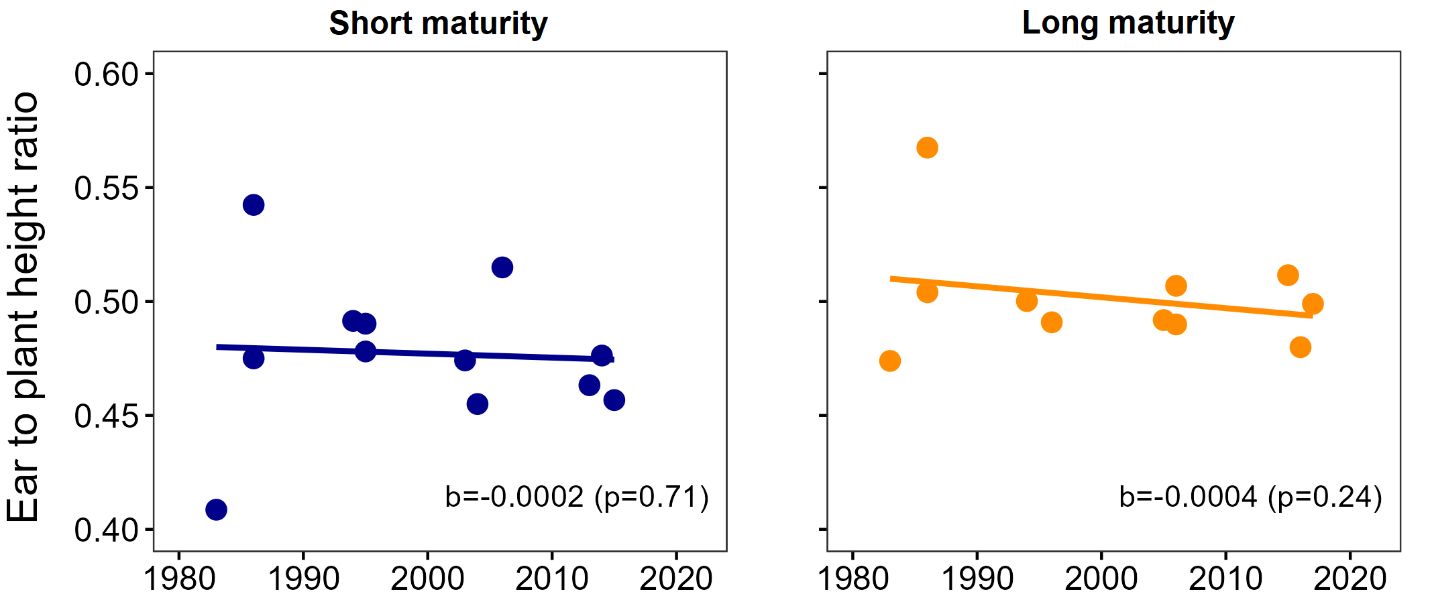


**Fig. S2.** Ear-to-plant height ratio in short maturity (103-day) and long maturity (111-day) hybrids released between 1980 and 2020. The points represent the average per hybrid across experiments. The values between brackets are the slope p-values.


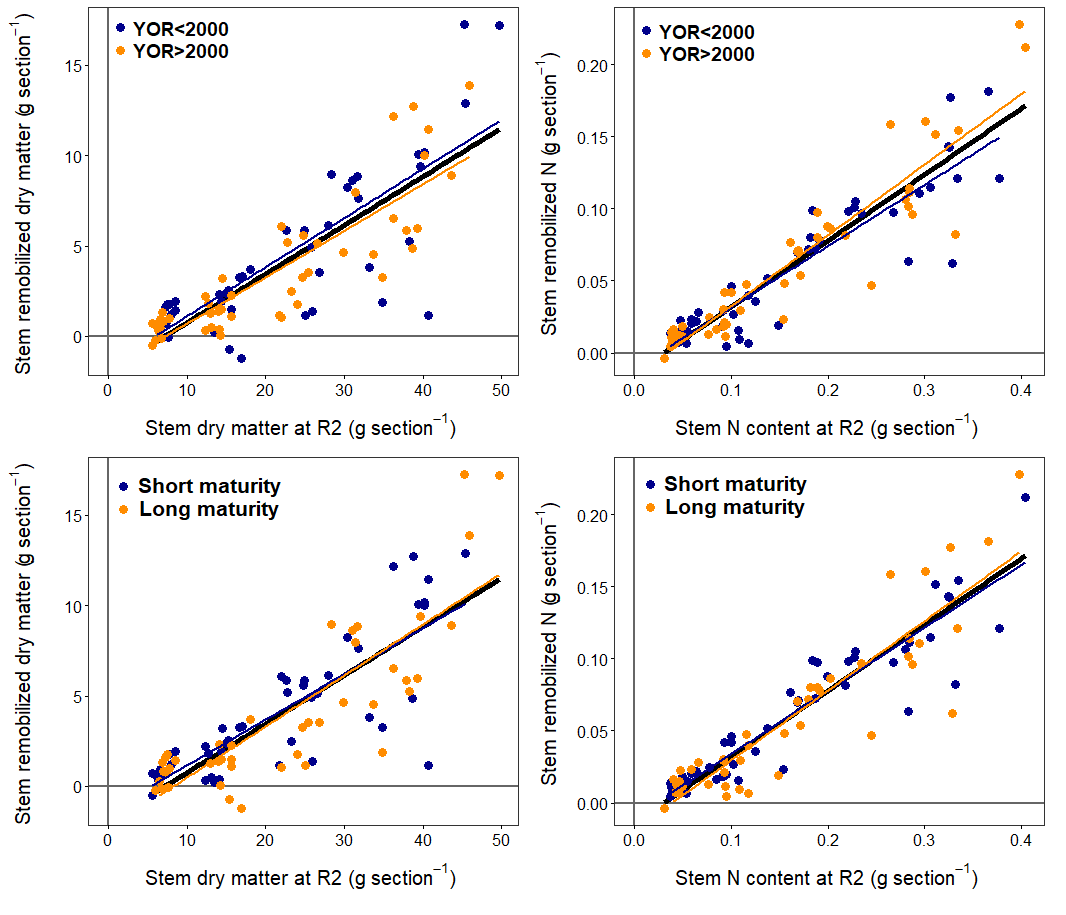


**Fig. S3.** Stem sections dry matter and N remobilization relationships across eras and relative maturities. Stem dry matter remobilization versus stem dry matter at R2 for hybrids released before and after 2000 (a); stem N remobilization versus stem dry matter N content at R2 for hybrids released before and after 2000 (b); stem dry matter remobilization versus stem dry matter at R2 for 103-day and 111-day relative maturity hybrids (c); stem N remobilization versus stem dry matter N content at R2 for 103-day and 111-day relative maturity hybrids (d).

**
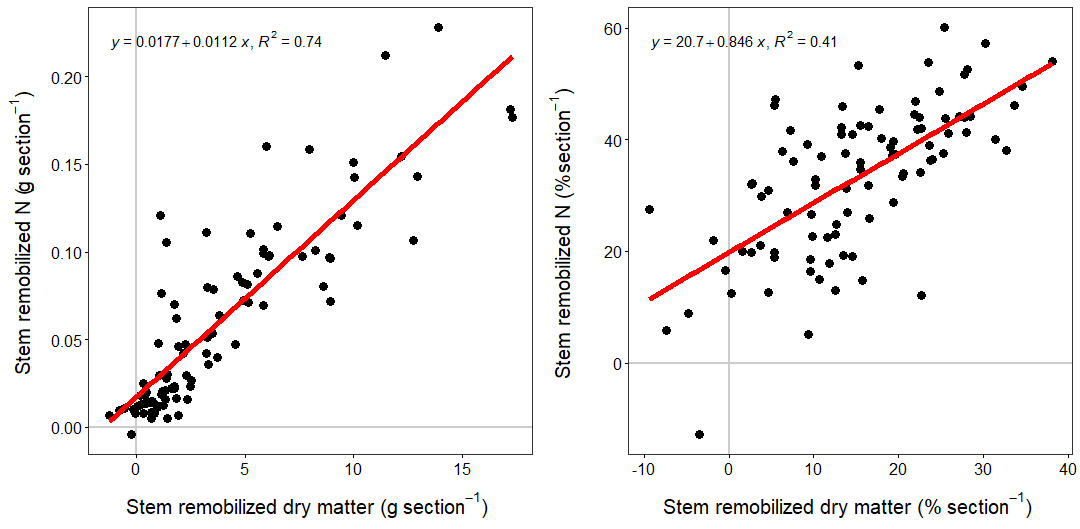
**

**Fig. S4.** Stem N remobilization versus stem dry matter remobilization. Absolute (left) and percentual remobilization (right).
